# A Pre–Post Evaluation of the Sohanjana Antibullying Intervention Among Students and Teachers in Rural Pakistan

**DOI:** 10.3390/healthcare14203550

**Published:** 2026-10-21

**Authors:** Sohni Siddiqui, Anja Schultze-Krumbholz

**Affiliations:** 1 Institute for Educational Research, Bergische Universität Wuppertal, 42103 Wuppertal, Germany; 2 Department of Educational Psychology, Technische Universität Berlin, 10587 Berlin, Germany; anja.schultze-krumbholz@tu-berlin.de

**Keywords:** Sohanjana Antibullying Intervention, rural public schools in Pakistan, teacher professional development, peer mentor training, pre–post experimental design

## Abstract

Introduction: Bullying and cyberbullying are widespread problems identified in numerous studies worldwide, including Pakistan. However, intervention plans specifically implemented in rural public schools remain limited. Purpose: This study aims to evaluate the outcomes of an antibullying intervention implemented in rural public schools in the Gilgit-Baltistan region of Pakistan, and to identify the opportunities and challenges involved. Methodology: The study used a quantitative pre–post experimental design and involved 25 teachers (6 teachers attended the full 32 h of training and 19 teachers attended partial training) from 16 institutions and 52 peer mentors from 7 schools who received 4 h of training. The dropout rate for teachers was different for every training, and details are mentioned in the study. In addition, the study sought to assess reductions in bullying and victimization three months after the trained teachers and peer mentors had implemented their learning. Data were collected from staff (*n* = 31 in 7 schools) and students (*n* = 35 in 4 schools) within the intervention sites. Results: Pre- and post-test results showed a significant increase in participants’ knowledge of bullying and strategies for dealing with the issue. Both victimization and perpetration as well as fear of being bullied at school were significantly reduced in student reports. However, no significant changes were observed in the data provided by staff. Pupils identified classrooms, particularly when teachers were absent, as the most common place for bullying to occur. Discussion: Key challenges in the training and implementation process included inadequate infrastructure, limited access to internet and electricity, and challenges in achieving consistent participation from teachers to apply their training. Some public-school teachers reported being transferred to other institutions shortly after the training, which prevented them from applying their learning. Many teachers who registered for the program did not attend the training sessions or the final presentation where they were expected to share their insights. Although many strategies were discussed during the training, most teachers focused only on giving awareness speeches and installing ‘bullying boxes’ as primary measures to tackle bullying. The challenge of collecting pre- and post-intervention data from schools was another major issue faced by the researchers. Recommendations: To improve the effectiveness of online programs in rural public schools, stronger policies, robust monitoring systems, improved infrastructure and better internet connectivity are essential. In addition, teachers need to be encouraged and supported to put their training in practice.

## 1. Introduction

Research from Pakistan shows that despite being a Muslim country where suicide is strictly forbidden and considered a great sin [1], cases of suicide have increased in recent years [2]. Gilgit-Baltistan is one of the regions where many suicide cases have been reported recently. Several studies have been conducted to investigate the causes and patterns of these incidents [3,4,5]. Among the reported causes, bullying stands out as a significant contributor, particularly among young people [2]. Despite the increasing number of suicides, government and NGO interventions have remained inadequate due to a lack of infrastructure and resources [6].

Bullying among school-aged children and adolescents is a significant public health and educational concern [7], particularly in settings where its psychosocial consequences and associated risk factors remain insufficiently investigated [8,9]. Gilgit-Baltistan is a remote mountainous region in Pakistan where research is limited and intervention studies remain sparse [10,11]. In response to reports of suicide [3,4,5] and bullying [2], the government and non-governmental organizations (NGOs) in Gilgit-Baltistan initiated awareness-raising efforts focused on mental health, suicidal ideation, and associated risk factors. Bullying is a serious social problem among school children in Pakistan, with significant psychological and social implications for the mental health of students [12]. Olweus (1993) define bullying as hostile, repetitive behavior inflicted by an individual or group [13]. Bullying and victimization are closely associated with negative academic outcomes such as poor performance and increased dropout rates [14,15]. Victimization is also a significant predictor of various physical and mental health problems such as anxiety, depression, psychosomatic symptoms, loss of appetite, stomach pain, dizziness, sleep disturbances and self-harm [7,15]. Bullying is a complex phenomenon [16] that can have serious consequences for victimized children, including suicidal ideation among the Pakistani students [17].

It has been estimated that approximately one in four Pakistani children experience bullying during their academic lives [18], indicating that bullying is a prevalent concern in Pakistani educational institutions [19,20]. In addition, Siddiqui and Schultze-Krumbholz (2023) found that approximately 98% of faculty members had either witnessed or received reports about the incidents of bullying at their institutions [9]. However, bullying has received relatively limited attention as a serious educational and social issue in Pakistan [21], and substantial national-level measures to address bullying in schools remain limited [22]. Several studies, including research conducted by the authors, have examined gaps in the existing response to bullying in Pakistani schools. Collectively, these studies have identified shortcomings in school-based antibullying efforts and emphasized the need for culturally and contextually appropriate approaches, including localized teacher training, counselling services, and policy-based strategies [19,21,23,24].

The existing literature also includes several preliminary initiatives and attempts to address bullying in Pakistani schools [25,26,27]. However, these initiatives have been characterized by methodological limitations, limited empirical evidence regarding their outcomes, or a lack of interventions specifically developed for the Pakistani sociocultural and educational context. For example, Hakim and Shah (2017) examined strategies used by teachers to manage bullying in primary schools in Haripur, Pakistan [25]. Their findings indicated that teachers emphasized establishing a safe physical environment, including communicating rules to students before engaging them in activities, as a strategy for managing bullying and other behavioural problems [25]. However, the study did not provide detailed guidance or systematically describe the specific steps required to establish and maintain a supportive and safe school environment. This highlights the need for more structured and contextually appropriate guidance for teachers, particularly in settings where systematic school-based antibullying programs remain limited.

To address the issue of bullying in schools, the Department of Educational Psychology at the Technical University of Berlin, the NGO Rupani Foundation and the Education Department of Gilgit-Baltistan collaborated on a project to train public school teachers. As reported by Salman et al. (2021), bullying is more prevalent in public schools than in private schools [28]. Therefore, public school teachers were specifically targeted for training. The training focused on bullying, its consequences and intervention strategies. The intervention was designed using a multilevel approach that targeted both teachers and peer mentors. Teachers were selected as a primary target because of their central role in identifying, preventing, and addressing bullying within school settings [29]. Effective teacher professional development should integrate theoretical and practical knowledge, authentic school-based situations, professional discussion, reflection, and opportunities for problem-solving [30,31]. Previous teacher-led interventions have also reported reductions in bullying-related outcomes following teacher-focused training [32,33]. The intervention also included peer mentors because bullying is often understood as a group process in which peers can assume different roles, including that of bystanders [34,35]. Bystanders represent a substantial proportion of students involved in bullying situations and may therefore be an important target for interventions seeking to influence peer norms and the broader school climate [35]. Accordingly, antibullying programs have increasingly incorporated bystander-focused components that seek to encourage supportive peer responses and challenge norms that may sustain bullying behaviour [36]. Including peer mentors alongside teacher training was therefore intended to address bullying at both the staff and peer levels rather than relying exclusively on teacher-led responses. The intervention was delivered as online training because online learning can provide greater flexibility and convenience while potentially reducing the costs and logistical demands associated with face-to-face training [37]. Participants can access training materials and sessions from different locations, which may be particularly useful in geographically dispersed or underserved settings where access to trained instructors may be limited. Online delivery can therefore facilitate the provision of professional development across locations that may otherwise be difficult for trainers to reach [37]. However, the practical feasibility of online delivery depends on local infrastructure, including the availability and reliability of internet connectivity, which is particularly relevant in rural settings.

This research study presents the results of the intervention, following the training of teachers and peer mentors who then implemented their learning in their respective schools. Additionally, the current study explored challenges related to online training programs for public school teachers and proposed potential solutions for future research. To researchers’ knowledge, this represents the first reported implementation of this type of antibullying training in the Gilgit-Baltistan region, which adds to the novelty and contextual relevance of the present study.

### 1.1. Problem Statement

Bullying is a prevalent concern in Pakistani schools [19,20], yet it remains insufficiently addressed [21], particularly in rural and underserved settings such as Gilgit-Baltistan. Gilgit-Baltistan is a region where numerous suicide cases have been reported, prompting several studies to investigate their causes and patterns [3,4,5], with bullying identified among the reported contributing factors, particularly among young people [2]. Existing antibullying initiatives in Pakistan are limited and often lack culturally and contextually adapted approaches [24], with little evidence from rural regions. There is therefore a need for structured interventions that engage both teachers, who play a central role in identifying and addressing bullying, and peers, who can influence bullying-related behaviours and norms. Online teacher training may provide a practical approach for reaching geographically dispersed schools [37], while peer-mentor models may complement teacher-focused efforts [24,36]. The present study therefore examines preliminary pre–post changes following the implementation of an online, contextualized, multilevel antibullying intervention involving teachers and peer mentors.

### 1.2. Originality, Rationale and Significance of the Study

This study is original in implementing a contextualized, online antibullying program in rural Pakistan that integrates teacher training with peer-mentor training. To our knowledge, it is the first such antibullying initiative reported in the Gilgit-Baltistan region. The intervention targets bullying at both the teacher and peer levels, with peer mentors specifically trained to promote active responses to bullying rather than passive bystanding [24]. This approach responds to the particular challenges of addressing bullying in rural and underserved settings. Teachers are central to identifying and responding to bullying, while peers shape bullying-related behaviors and social norms [24,36]. Given geographical dispersion and limited resources, conventional in-person professional development is often impractical. Online delivery therefore offered a feasible way to extend teacher training across multiple schools, while peer-mentor training complemented the teacher-focused component. Nonetheless, implementing online training in rural contexts may be constrained by factors such as internet connectivity, attendance, and available resources. Beyond changes in bullying-related outcomes, the study’s significance lies in providing initial insights into delivering a multilevel antibullying intervention under real-world conditions in a rural, underserved educational setting in Pakistan. Practically, the findings may guide the design of teacher professional development, peer-mentor strategies, and methods to strengthen implementation and fidelity. The implementation experience also highlights key considerations for school leaders and education authorities, including teacher participation, administrative support, digital infrastructure, and access to professional development. More broadly, the study adds preliminary evidence to the sparse literature on antibullying interventions in rural Pakistan and may inform future controlled studies on their effectiveness and long-term sustainability.

### 1.3. Research Questions

1. Does participation in the culturally sensitive antibullying intervention improve participants’ knowledge of bullying and appropriate intervention strategies in rural schools?
2. Is participation in the culturally sensitive antibullying intervention associated with reductions in bullying perpetration and victimization in rural schools?
3. Is participation in the culturally sensitive antibullying intervention associated with changes in school climate and related outcomes, including teachers’, students’, and peers’ attitudes toward intervening in bullying, students’ liking of school, fear of being bullied, and empathy toward victims of bullying?
4. What are the main areas where bullying and victimization occur within rural school settings?
5. What are the main challenges and barriers associated with implementing an online antibullying intervention in rural schools?

## 2. Literature Review

### 2.1. Status of Bullying Interventions in Pakistan

Bullying has been reported as a prevalent issue among adolescents in the Global South, Global North, and South Asian regions, including Pakistan [9,17]. This widespread problem has led to numerous negative outcomes, both internationally and for Pakistani students, such as suicidal ideation [17], adverse effects on mental health [38], emotional well-being, social, and academic functioning [23]. It has also contributed to psychosocial issues [39], disturbances in personal and social life [21], frequent school absenteeism and dropout [23], communication problems [40], and a decline in overall well-being [41]. Several studies have documented bullying and victimization in Pakistan. Naveed et al. (2020) found that 26.6% (*n* = 615) of students (mean age = 14.63 years, N= 2325) reported experiencing bullying at school, while 17.9% (*n* = 415) reported being bullied outside of school [23]. Additionally, 18.6% (*n* = 430) of students admitted to engaging in bullying behavior at school, and 16.3% (*n* = 376) admitted to perpetrating it outside of school. The study also revealed a strong association between past experiences of being bullied and later becoming a bully [23]. Regarding older students, Ayub et al. (2022) reported that nearly 70% of postgraduate trainees experienced bullying within their institutions [42]. Similarly, Ahmer et al. (2008) reported that 52% of students from medical educational institutions in Pakistan had experienced bullying, with verbal bullying being the most common form (57%) [43]. Mirza et al. (2020) found that 58.7% of university students had experienced various forms of cyberbullying [44]. Likewise, Saleem et al. (2021) found that around 90% of university students were subjected to cyberbullying [19]. Another study by Hameed and Ismail (2024) reported that 60.5% (*n* = 121) of female university students encountered cyberbullying, while 22% (*n* = 44) had no such experience [45]. Accordingly, Siddiqui and Schultze-Krumbholz (2023) found that approximately 98% of faculty members had either witnessed or reported incidents of bullying at their institutions [9]. Despite the high prevalence of bullying reported in numerous cross-sectional studies, intervention strategies remain inadequate and require greater attention from authorities [46]. The Sohanjana Antibullying Intervention was specifically designed for institutions in Pakistan, taking cultural and contextual factors into account [22]. Its initial implementation showed significant success in enhancing teachers’ and students’ knowledge and understanding of bullying, its consequences, and effective strategies [29,47,48]. It was also partially successful in reducing fifth grade students’ victimization in institutions where it was applied [29]. In the present study, the intervention was delivered online to rural areas of Pakistan, where similar evidence-based research is lacking, aiming to address this gap.

### 2.2. Scope of Online Training in Pakistan

Pakistan is a developing country where teacher training is still essential, particularly in addressing bullying issues [46,49]. To reach a wider audience, online learning has become increasingly common, especially after the COVID-19 pandemic [50]. Internationally, online in-service training is considered essential and plays a pivotal role in the professional development of teachers [51]. With this in mind, an online antibullying intervention was developed for Pakistani teachers in 2023 by international researchers [22]. Online training, conducted entirely over the internet, incorporates various multimedia elements such as images, audio, video, and web links [52]. One of the key advantages of online learning courses is flexibility, which also enhances convenience and reduces costs. Trainees can conveniently access the courses from anywhere and, in some cases, at flexible times [37]. This makes online learning particularly beneficial in areas that are difficult for instructors to reach, as digital platforms facilitate instruction regardless of location constraints [37].

### 2.3. Theoretical Framework Underpinning the Sohanjana Antibullying Intervention

The Sohanjana Antibullying Intervention was developed from a multidimensional theoretical framework addressing individual, interpersonal, and environmental factors associated with bullying [24]. Five complementary theoretical perspectives informed the development of its training modules: Bronfenbrenner’s Ecological Systems Theory, the Bystander Intervention Model, Social Capital Theory, Theory of Humiliation and the Theory of Planned Behaviour. The theoretical foundations of Sohanjana Antibullying Intervention informed both the selection of intervention targets and the expected mechanisms through which change might occur. Bronfenbrenner’s Ecological Systems Theory provided the overarching framework for the multilevel design by conceptualizing bullying as a behaviour shaped by individual, peer, teacher, school, and broader environmental influences [53]. This perspective supported the inclusion of both teacher training and peer-mentor training rather than focusing on students as individual perpetrators or victims alone.

The Bystander Intervention Model provided the primary theoretical basis for the peer-mentor component [54]. The model proposes that effective intervention requires individuals to recognize an incident, interpret it as requiring action, accept responsibility, know how to respond, and take appropriate action. Peer-mentor training therefore aimed to strengthen students’ awareness, responsibility, and practical skills so that they could move from passive observation toward constructive responses to bullying [36,55]. Social Capital Theory complemented this approach by emphasizing the importance of peer relationships and social networks in shaping students’ experiences and responses to bullying.

Social Capital Theory and the Theory of Planned Behaviour particularly informed the teacher-training component. Social Capital Theory emphasizes the importance of supportive relationships and social networks, highlighting the role teachers can play in creating supportive school environments and strengthening positive peer relationships [56]. The Theory of Planned Behaviour further informed training content addressing teachers’ attitudes, perceived norms, and perceived behavioural control regarding bullying prevention and response [57]. Together, these perspectives suggest that increasing teachers’ knowledge, awareness, and confidence may support more consistent recognition and management of bullying.

Lindner’s Theory of Humiliation provided an additional perspective for both teacher and peer training by emphasizing dignity, recognition, and respect in interpersonal relationships [58]. This perspective informed activities addressing the social and emotional consequences of bullying and promoting respectful and assertive responses to humiliating interactions.

Together, these theories suggest several interconnected mechanisms of change: improved teacher knowledge and perceived capacity to respond to bullying, greater awareness and responsibility among peer mentors, more supportive peer relationships and norms, and increased recognition of bullying and appropriate responses [24]. These theoretical perspectives also provide a framework for interpreting the study findings from previous implementations. For example, improvements in training knowledge can be considered in relation to the teacher-focused mechanisms [48], while changes in student-reported bullying in relation to the proposed peer, relational, and ecological mechanisms [29].

### 2.4. Sohanjana Antibullying Intervention-Teachers and Peer Mentors Training Program

Preventing bullying in educational institutions through teachers’ professional development has been widely supported by international research and subsequent programs, such as the P.E.A.C.E Pack [59,60], ViSC (Viennese Social Competence Program) [30], Relationships to Grow (RPC) [61], and Media Heroes [62], and these interventions have shown considerable success. Hektner and Swenson (2012) highlighted that teacher intervention significantly reduces bullying and victimization and improves the overall school environment [63]. However, successful interventions require both specific individual teacher characteristics and supportive contextual factors [64].

Recognizing the need for cost-effective teacher-led programs, especially in low- to middle-income countries like Pakistan, the Sohanjana Antibullying Intervention was developed [22]. This program addresses the gap in teachers’ understanding of antisocial behaviors by offering 32 h of training across eight modules, covering both proactive and reactive approaches to managing bullying. For more details on this teachers’ development program, see Siddiqui et al. (2023) [22]. To increase the impact of the program, a team of peer mentors was also introduced to support teachers. A study by Siddiqui and Schultze-Krumbholz (2023) in Pakistan revealed that only 10% of peers reported bullying incidents, while most witnesses either ignored the situation or distanced themselves from it [9]. To address this passive bystander behavior, the Sohanjana program included a 4-h training for students, alongside the 32-h teacher training. This peer training was designed to empower students as leaders and advocates for antibullying efforts in their school communities. While bystander training is a key component of many international interventions [65], the Sohanjana Antibullying Intervention is the first program in Pakistan to focus on training peer mentors and transforming passive bystanders into active participants in preventing bullying. For a detailed discussion on this peer training initiative, see Siddiqui and Schultze-Krumbholz (2023) [47].

Findings from previous implementations of the Sohanjana Antibullying Intervention showed a mixed pattern. Teacher training was associated with increased awareness of bullying prevention strategies and its consequences [48], while staff reports indicated decreases in verbal, social, and physical bullying that did not reach statistical significance [48]. In contrast, student reports from rural schools showed a statistically significant reduction in physical bullying [29], while decreases in victimization and perpetration were observed across grade levels, with a statistically significant reduction in victimization among Grade 5 students [29]. The intervention also showed improvements in students’ bystander roles, which was particularly relevant given the limited evidence of peer-led antibullying responses in the Pakistani context [29]. However, previous implementation was based on a relatively small sample involving four schools, including one control and three intervention schools [29,48], limiting the generalizability and precision of the findings. The observed variations across outcomes also highlighted the need for further investigation using larger samples and broader implementation contexts. Accordingly, the present study was designed to extend this work by implementing the intervention across a larger number of schools in a region where structured antibullying programs remain limited and by examining pre–post changes following implementation. The study also aimed to provide further information that could inform future longitudinal research examining whether observed changes are maintained over time. This research study aims to assess the overall improvement in the school climate of public rural Pakistani schools from the perspectives of multiple stakeholders. In addition to staff, students also provided pre- and post-intervention data to determine if the implementation had a significant impact on reducing bullying and victimization. The study also evaluated whether the participating teachers and peer mentors in training, showed an increase in their knowledge about bullying and its prevention.

The current study aims to evaluate the effectiveness of an online antibullying training program in rural regions of Pakistan, focusing not only on its impact but also on the benefits and challenges associated with implementing online training in a region that lags behind others in several aspects. Given that such programs are rarely designed and executed in these areas, the study also provides potential solutions for delivering similar trainings in the future.

## 3. Research Methodology

### 3.1. Research Design

A single-group pre–post design was employed to compare the selected outcomes before and after implementation of the intervention. Pre-intervention measurements were collected prior to the implementation, and the same outcomes were assessed again following implementation. The pre–post comparisons were used to examine changes in participants’ outcomes over the course of the intervention. As the study did not include a control or comparison group, the observed changes should be interpreted as pre–post differences rather than evidence of a causal effect of the intervention. It should be noted that data were collected from both trained teachers, who participated in 32 h of online training, and peer mentors, who completed 4 h of online peer-mentor training. These participants were assessed regarding improvements in their knowledge. In addition, untrained staff members and students requested to contribute information on bullying incidents, allowing bullying reports to be compared before and after the implementation of the intervention by the trained teachers and peer mentors. Inferential analyses were conducted using SPSS (version 27).

### 3.2. Training Format

The training was delivered through an online format using the Zoom platform, although participants attended the sessions from designated physical location. Teachers from different institutions gathered at a central location arranged by the study organizers, primarily because of internet connectivity limitations at the participating institutions. To facilitate access to the online sessions, the organizers provided a laptop connected to a large screen, which enabled the trainers to deliver the training content and interact with the participating teachers in real time through Zoom. The teachers used their own personal devices, including laptops, tablets, and mobile phones, to complete the training activities. The training content and activities were specifically designed for online delivery. These included interactive case-based activities, such as reading case scenarios and proposing appropriate intervention strategies. The teacher training also incorporated role-play activities, during which teachers performed different scenarios in front of the camera and interacted in real time with the trainers through Zoom and other participants. For the peer-mentor training, participating schools used their computer laboratories, where students joined the four-hour training session through Zoom. Thus, although teachers were physically gathered at a central location for logistical and connectivity reasons, the training itself was delivered online through Zoom. The instructional content, trainer–participant interactions, and training activities were conducted through the online platform, while the physical locations primarily provided participants with a suitable setting and reliable internet access to engage in the sessions. Accordingly, the intervention was classified as an online training program.

The planned teacher training comprised 32 h delivered across eight modules. Attendance at all eight modules was considered full exposure to the face-to-face training, whereas attendance at fewer modules represented partial exposure. All registered teachers were provided with the training presentations and module books; however, independent review or use of these materials by teachers who missed training sessions was not monitored and therefore could not be considered equivalent to attendance. For timeline details of the study refer to Figure 1.

### 3.3. Sampling Procedure

#### 3.3.1. For Teacher Training

To recruit schools for the program, the researchers contacted the Rupani Foundation (https://rupanifoundation.org/, accessed on 31 March 2024), a non-governmental organization in Pakistan dedicated to community-driven development solutions that address development challenges and reach vulnerable populations. Following a successful online meeting with the researchers, the Foundation collaborated with the Department of Education, Government of Gilgit Baltistan. After a productive meeting and approval of the proposal, the Department of Education issued official letters of invitation and brochures detailing the dates, times, modules and objectives of the program to a wide range of schools. As a result, 16 government schools in different regions of Gilgit Baltistan expressed interest and submitted nominations of teachers who were either interested or nominated by heads of institutes to attend the training. Convenience sampling was used, with teachers from primary, secondary and higher secondary government schools enrolled in the training. A total of 43 participants registered for the training; however, only 6 (14%) attended all sessions, while 16 (37%) did not attend a single session. The remaining participants attended the sessions partially. Detailed information on participant attendance is presented in Figure 2. As the post-training test results were calculated using end-of-session assessments, variations in participant numbers were observed in different sessions. Notably, 25 participants (Male = 13, Female = 12, M_age_ = 40.0) attended the first session, during which demographic information was collected, which is reported in Table 1.

#### 3.3.2. For Peer Mentor Trainings

The primary objective of these training sessions was to equip students with the essential skills to encourage pro-social bystander behavior, train peer mentors to identify victims and support them in self-advocacy, and to assist perpetrators in modifying their behavior. A total of 88 registrations for the peer mentor training were submitted by trained teachers; however, only 52 (59%) students participated in the peer mentor sessions. When staff members were asked about the reasons for participants’ dropout, they reported that some peer mentors were engaged in other school activities and were therefore unable to attend because of scheduling conflicts. Other participants were absent from school without providing a documented reason. Regarding participant selection, schools used a combination of voluntary participation and nomination by school staff. It was communicated to participating schools that peer mentors should be recruited from the secondary school level, with participation from each school limited to between 5 and 15 peer mentors. For the demographics of peers who successfully completed the training refer to Table 1.

#### 3.3.3. Non-Trained Staff and Students

Before and after the intervention was implemented, trained teachers were asked to collect data from their respective schools, involving both staff members and students. An online link for pre- and post-intervention data collection was shared with the trained teachers for this purpose. Before the intervention and data collection, trained staff were explicitly instructed to inform parents about the intervention and the associated data collection procedures. The participating schools prepared an information and consent letter on their official school letterhead and distributed it to the parents/guardians of the participating students. Parents/guardians were asked to acknowledge their consent by signing the letter and returning it to the school. Thus, parental permission was obtained through a documented written process rather than solely through students’ confirmation. As an additional safeguard, before data collection at each assessment phase, students were again inquired that their parents/guardians had been informed about the study and data collection. Students were required to confirm this by selecting “yes” on the online questionnaire before they could proceed to the study questions. This procedure provided an additional opportunity for students to confirm parents’ willingness to participate at each data-collection phase. Only students with parental consent were allowed to complete the form. It should also be noted that 69 students provided data during the pre-intervention data collection phase, whereas 58 students provided data at post-intervention assessment. However, for hypothesis testing, only students who provided data at both the pre- and post-intervention stages (*n* = 35) were included in the analysis, representing an attrition rate of approximately 49.3%**.** In case of the staff members, the form proceeded only after the staff members gave their consent to participate. In this way, informed active consent (opt-in) was obtained from all participants. The sampling technique was convenience/purposive sampling. For staff members, the form included a detailed introduction explaining the purpose of the study.

### 3.4. Instruments

The study used a variety of instruments at different stages to assess program outcomes.

#### 3.4.1. Questionnaires Designed to Measure Knowledge Improvement

To assess the impact of the training on improving the understanding of bullying and related issues, the researchers developed a series of declarative knowledge questions. Pre- and post-tests were administered for each module to both teachers and peer mentors. The tests comprised open- and closed-ended questions, with a maximum score of 8 points, and were accompanied by a corresponding scoring rubric to assess changes in knowledge. The same set of questions was used for both the pre- and post-assessments. These questions pilot -tested and revised [22,49,66], and subsequently used during the implementation of the final intervention [48]. The first author used a scoring rubric that had been previously tested in earlier studies and subsequently discussed the scores with the second author. Both authors reviewed and agreed on the final scores before proceeding with hypothesis testing. As an example, questions from Module IV, which focused on building empathy among students, included the following:

Close-ended question: When bystanders defend the target of bullying and intervene, the bullying stops within ………… more than half the time.

Options:

- 10 min
- 30 min
- 10 s
- 30 s

Open-ended question: Suggest two ways to develop or enhance empathy among students/teenagers for bullying intervention.

#### 3.4.2. Pre–Post Bullying Prevalence (Students’ Report)

The study also aimed to investigate the reduction in bullying and victimization following the intervention and improvements in various aspects of the school environment. A total of 30 items were used. They pertained to aspects such as satisfaction with the school environment, comfort level at school, empathy for victims, and willingness to help and stand up against bullying. For this purpose, questions from The Revised Bully/Victim Questionnaire (RBVOQ) [67] were used. Variables a and b (Victimization and Perpetration) consisted of 10 items each, whereas the remaining variables were assessed using single-item measures. The Likert scale used in this tool varies for different statements and is explained below:

- Victimization: 10 items on a 5-point Likert scale (1 = “It hasn’t happened to me in the past couple of months” to 5 = “It happened several times a week”), Cronbach’s α = 0.889 [20] (Example Statement: Other students left me out of things on purpose, excluded me from their group of friends, or completely ignored me)
- Perpetration: 10 items on a 5-point Likert scale (1 = “I haven’t bullied another student(s) at school in the past couple of months” to 5 = “I bullied several times a week”), Cronbach’s α = 0.937 [20] (Example Statement: I kept him or her out of things on purpose, excluded him or her from my group of friends or completely ignored him or her)
- Institutional Staff Attitude: A single-item variable using a 5-point Likert scale (1 = “Almost never” to 5 = “Almost always”). (Statement: How often do the teachers or other adults at school try to put a stop to it when a student is being bullied at school?)
- Students Attitude: A single-item variable using a 5-point Likert scale (1 = “Almost never” to 5 = “Almost always”). (Statement: How often do other students try to put a stop to it when a student is being bullied at school?)
- Parents/Guardian Attitude: A single-item variable using a 5-point Likert scale (1 = “Almost never” to 5 = “Almost always”). (Statement: Has any adult at home contacted the school to try to stop your being bullied at school in the past couple of months).
- Class Teacher Attitude: A single-item variable using a 5-point Likert scale (1 = “Little or nothing” to 5 = “Much”). (Statement: Overall, how much do think your class (home room) teacher has done to counteract bullying in the past couple of months?)
- Liking towards school: A single-item variable using a 5-point Likert scale (1 = “I dislike school very much“ to 5 = “I like school very much“). (Statement: How do you like school?)
- Tendency towards bullying: A single-item variable using a 6-point Likert Scale (1 = Yes, 6 = Definitely No). (Statement: Do you think you could join in bullying a student whom you didn’t like?)
- Reaction towards Bullying: A single-item variable using a 6-point Likert Scale (1 have never noticed that students my age have been bullied, 2 = I take part in bullying, 6 = I try to help the bullied student in one way or another) (Statement: How do you usually react if you see or understand that a student your age is being bullied by other students?)
- Fear of getting bullied: A single-item variable using a 6-point Likert scale (1 = “never“ to 6 = “very often”). (Statement: How often are you afraid of being bullied by other students in your school?)
- Empathy: A single item variable using a 4-point Likert scale (1 = “that is probably what he or she deserves“ to 4 = “I feel sorry for him or her and want to help him or her“). (Statement: When you see a student, your age being bullied at school, what do you feel or think?)
- Place of bullying: a single item on where students have been bullied; eleven different locations were provided as options, along with an additional option for students who wished to report a different location.

#### 3.4.3. Pre–Post Bullying Prevalence (Staff Report)

To assess changes in bullying incidents before and after the program, a questionnaire was adopted from previous research to measure cyber, social/emotional, physical and verbal forms of bullying. This questionnaire had been used in several previous studies [9,48], thus eliminating the need for factor analysis. It should be noted there are no reverse coded questions in the questionnaires used. Further details are provided below.

- Cyberbullying was assessed with an adapted version of the Berlin Cyberbullying-Cybervictimization Questionnaire (BCyQ) by Schultze-Krumbholz and Scheithauer (2011) [68]. A total of 17 items with a 5-point Likert scale (1 = never to 5 = frequently) (Cronbach’s alpha = 0.931, AVE = 0.549) [22] were used in the study. An example is: “Others spread embarrassing, insulting or humiliating video clips/photos of student without permission on the Internet or by mobile phone”.
- Emotional/Social bullying was measured with 9 items developed by Doğruer (2015) [69]. A five-point Likert scale was used (1 = never to 5 = frequently) (Cronbach’s alpha = 0.901, AVE = 0.557) [22]. An example is: “Some student(s) tell lies and stories about others students to make them look bad”.
- Verbal bullying was assessed using adapted and contextualized items from different studies [69,70,71]. Seven items with a five-point Likert scale (1 = never to 5 = frequently) were used (Cronbach’s alpha = 0.895, AVE = 0.614) [22]. An example is: “Some student(s) has insulted or said nasty words to other”.
- Physical bullying was measured with seven items adapted and contextualized from different studies [70,71,72] on a five-point Likert scale (1 = never to 5 = frequently) (Cronbach’s alpha = 0.915, AVE = 0.663) [22]. An example is: “Some student(s) have thrown things at another student or hit with object for physical abuse”.

## 4. Data Analysis and Results

The quantitative data was analyzed using SPSS version 27. After confirming the reliability of the questionnaires, the Wilcoxon Signed Rank Test was used to measure differences in participants’ knowledge before and after the training. A Wilcoxon signed rank test was also used to assess reductions in bullying and victimization at participating schools, improvements in stakeholders’ attitudes toward the bullying intervention, and various aspects of the school climate based on student and staff reports. The Wilcoxon Signed Rank Test is a nonparametric test, meaning it does not assume a normal distribution of the data. This is especially useful when the sample size is small and the assumption of normality cannot be easily verified. As the sample size was small, we preferred to use this test for the assessment of pre–post differences. Effect sizes were calculated using (r = |Z|/√N), where (Z) is the standardized Wilcoxon signed-rank test statistic and (N) is the number of paired participants included in the respective analysis. Thus, for a pre–post analysis, the two measurements contributed by each participant constitute one paired observation, and (N) corresponds to the number of participants with complete pre- and post-test data.

It should be noted that blinding was not feasible in the present study because of the nature of the behavioral teacher-training intervention. Participants were aware of their participation in the intervention, and data collectors involved in the intervention were also aware of the study procedures. This awareness may have introduced expectancy or social-desirability effects, particularly for self-reported outcomes. Consequently, the observed pre–post changes should be interpreted cautiously and cannot be attributed solely to the intervention. Future studies could reduce this potential source of bias by using independent, blinded data collectors where feasible and by incorporating multiple sources of outcome assessment.

### 4.1. Pre–Post Training Results on Knowledge Improvement (Trained Teachers and Peer Mentors)

One of the main objectives was to determine whether teachers and peer mentors who completed the training showed improvement in their understanding of key bullying-related concepts, including its definition, types, roles, consequences, and effective intervention strategies discussed during the sessions. A Wilcoxon signed-rank test was conducted using SPSS version 27 for the pre–post evaluation. The results revealed a significant positive improvement among teachers and peer mentors who completed the training, with moderate (r > 0.3) to a strong effect size (r > 0.5) (refer to Table 2). To account for the increased risk of Type I error associated with multiple comparisons, Holm’s sequential Bonferroni procedure was applied to the nine pre–post comparisons of training-related knowledge (eight teacher training modules and one peer-mentor training). The adjusted *p*-values were used to determine statistical significance while controlling the family-wise error rate at α = 0.05. All nine comparisons remained statistically significant after the adjustment. This indicates that the intervention has the potential to enhance teachers’ and peer mentors’ knowledge about bullying and equip them with effective strategies for addressing it.

### 4.2. Pre–Post Intervention Results in Reducing Bullying and Victimization (Students Report)

One of the main objectives of the study was to assess the impact of the training on reducing bullying and victimization within the institution where the intervention was implemented. For this purpose, data were collected from students at the participating institution. Unfortunately, despite considerable efforts, substantial data could not be gathered due to internet and electricity issues, and in some cases, a lack of support from the management further hindered the process.

Reliability statistics for Revised Bully/Victim Questionnaire (RBVOQ; Olweus, 1996) are presented in Table 3 [67]. A total of 69 students participated in the pre-test and 58 in the post-test, but only 35 students (M age = 14.4 years) completed both pre- and post-test assessments (For demographic details refer to Table 1). Paired analyses were conducted using the 35 students who provided both pre- and post-test data. A comparison between students who completed both assessments and those who did not complete both assessments was not conducted because the available data did not permit a sufficiently informative comparison. This should be noted that 35 matched student observations were nested within only four schools and therefore cannot be considered equivalent to 35 observations obtained from 35 independent schools. Consequently, the statistical results should be interpreted cautiously, as conventional analyses that do not account for clustering may underestimate uncertainty. Due to the small sample size, factor analysis was not conducted, as the instrument has already been validated in two previous studies in Pakistan [20,29]. A Wilcoxon signed rank test revealed a significant reduction in both bullying perpetration and victimization following the intervention, with medium to strong effect sizes (see Table 3). For the student-reported bullying outcomes, researchers applied Holm’s sequential procedure to the two pre–post comparisons of victimization and perpetration. Statistical significance was evaluated at α = 0.05 using the Holm-adjusted *p*-values. Both comparisons remained statistically significant after adjustment for multiple comparisons. In addition to evaluating bullying and victimization, the researchers also assessed the impact of the training on improving the attitudes of institutional staff, parents, and students, however no significant results were observed (see Table 4). Similarly, the researchers explored additional aspects of school life, such as students’ liking for school, improvements in empathy towards victims, fear of being bullied, and their tendency to engage in bullying. While no significant differences were observed in most of these areas, there was a notable reduction in students’ self-reported fear of being bullied at school (see Table 4) with a large effect size (r = 0.5). Fear of getting bullied remains statistically significant after Holm correction (adjusted *p* = 0.045). This indicates the program’s positive outcomes in creating a bullying free zone in schools, where students may have felt safer after the intervention.

### 4.3. Bullying Location

During the pre-intervention data collection, students were also asked to identify the locations where they most frequently witnessed bullying. Students were given the option to select multiple locations. Of the 35 students included in the analysis, 22 did not indicate any location, 11 selected one location, and 2 students selected two locations. Thus, a total of 15 location selections were recorded. Because students could select more than one location, the reported frequencies represent the number of location selections rather than the number of respondents. This question was administered only at the pre-intervention stage, as its purpose was to identify locations where bullying was more frequently observed and communicate this information to teachers so that they could increase supervision in those areas. When asked about the most common places within their educational institution where bullying incidents typically occur, students gave the following responses: The majority identified classrooms when the teacher is absent (*n* = 7) as the most common setting, followed by classrooms even when the teacher is present (*n* = 2), routes to and from school (*n* = 2), the playground (*n* = 1) and the lunchroom (*n* = 1). A total of 2 students also reported that they witnessed bullying some other place which are not given in common options.

### 4.4. Pre–Post Intervention Staff Reports

The aim of the study was to evaluate the impact of the training on reducing bullying within the institution where the intervention was implemented, from the perspective of the staff. To achieve this, data was collected from staff at the participating institution, with reports collected to assess changes in different forms of bullying, including cyber, social, verbal and physical. Since the sample size was very small, a non-parametric Wilcoxon signed rank test was used to analyze the data and identify any differences. Factor analysis was not conducted due to the small sample size, although the same part of the instrument was validated in a previous study [9]. Reliability statistics for the instrument used in both the pre- and post-intervention assessments are presented in Table 5. A total of 31 staff members provided both pre and post data (demographic details are provided in Table 1). The Wilcoxon signed rank test showed no significant reduction in any form of bullying (see Table 5), although there was a slight reduction in emotional/social bullying. For the remaining forms of bullying, there was a slight increase in the post-intervention scores, but again increase was not significant. These findings should be interpreted cautiously and should not be taken as evidence that the intervention had no effect. The staff sample was relatively small (*n =* 31), which limited statistical power and reduced the precision with which changes could be estimated. The observed effect sizes were small across the four outcomes, ranging from r = 0.021 for physical bullying to r = 0.217 for cyberbullying. Thus, the findings provide limited evidence of statistically reliable change in staff-reported bullying within the present sample, but they cannot establish the absence of an intervention effect. Larger samples and controlled designs are required to determine whether the observed patterns represent meaningful changes in staff-reported bullying.

### 4.5. Challenges During the Intervention Training

One of the objectives of the study was to identify the challenges faced by researchers during the training, implementation, and evaluation of the program. The implementation challenges reported in the manuscript were identified from multiple sources generated during the implementation of the intervention, including attendance and participation records, researcher observations and field notes, feedback and reports provided by participating teachers and staff, communication with participating schools during implementation, and observations made during the delivery and completion of the intervention activities. These information sources were collected throughout the implementation period by the research team and were used to document practical difficulties encountered during intervention delivery and participation.

The challenges were not identified using a pre-specified qualitative coding framework, nor was a formal qualitative analysis conducted. Instead, the information was reviewed after implementation to identify recurring practical and contextual difficulties that affected intervention participation, delivery, or completion. The identified observations were subsequently grouped into broader descriptive categories based on their common characteristics, such as participant availability and attendance, technological or logistical difficulties, and school-level implementation conditions.

While the results were positive, they could have been more robust if the issues had been addressed prior to implementing similar trainings in the future. A summary of the challenges encountered during the intervention program is provided in bullet points and discussed in detail in the discussion section.

#### 4.5.1. Lack of Supportive Infrastructure

The online training faced numerous challenges from day one, primarily due to internet connectivity issues. Initially, the plan was to conduct the training at the respective institutions, but due to a lack of internet access and devices, the collaborating organizations decided on a central venue. However, even at the venue, the internet connection remained unstable. A similar problem arose during the data collection process, where there was no support system in place for gathering data through online platforms, further complicating the process. As recommended by Siddiqui and Schultze-Krumbholz (2026), ensuring the long-term effectiveness of the intervention required an additional data collection phase six months after implementation [48]. However, due to persistent internet issues and challenges in obtaining staff and students responses during the post-intervention data collection phase, the plan for further data collection was canceled. Researchers anticipated an even lower response rate in the next phase, making continuation unfeasible.

#### 4.5.2. Limited Participation and Responsiveness

Although some teachers showed enthusiasm and commitment to attending sessions and implementing their learning, the majority were unable to attend all sessions and missed sessions due to logistical issues. In addition, only few schools (7 out of 16) supported the training of peer mentors after the end of the program and even fewer continued to the data collection phase (4 out of 16). To keep participants motivated, the training was offered free of charge, with certification on completion. Lunch and refreshments were also provided during the training days. Despite these incentives, many teachers had logistical or travel problems and were only able to attend a few sessions or only part of the time. Another challenge faced by the researchers was difficulty in accommodating participants’ availability, particularly for female teachers. The discussion section discusses possible social norm and reasons for women’s reluctance to actively participate in such programs. Several participants reported scheduling constraints related to household responsibilities and childcare, which affected their ability to attend sessions held later in the day. As the researchers were based in Germany and had originally planned to start the sessions early in the morning at around 8 am (German time), this schedule was not well received by the trainees. As a result, the researchers adjusted the schedule by moving the start time to 6 a.m. to better accommodate the needs of the participants. However, despite the time change, many teachers were still unable to attend all the sessions and missed parts of the training. These attendance patterns highlight the importance of considering participants’ local schedules and contextual responsibilities when planning the timing of remotely delivered professional development programs.

#### 4.5.3. Lack of Implementation and Intervention Fidelity

The Sohanjana Antibullying Intervention was an intensive 32-h training program for teachers and 4-h training program for peer mentors that covered various strategies throughout the sessions (see Siddiqui et al., 2023 and Siddiqui & Schultze-Krumbholz, 2023 for details) [22,47]. However, during the final presentation, an essential component of the program where teachers were expected to share their experiences and how they implemented the intervention, it became evident that many strategies were not applied. Intervention fidelity is an important consideration when interpreting the findings. Although teachers received training on multiple intervention strategies, implementation varied across schools, and some teachers implemented only selected components. Consequently, the observed outcomes should be interpreted as reflecting the effects of the intervention under conditions of partial and variable implementation rather than under full adherence to the intended intervention protocol. Most teachers focused primarily on raising awareness through morning assemblies and installing bully boxes for reporting incidents. Several teachers failed to attend the final presentation, offering last-minute excuses, while some did not notify the organizers at all. In some cases, teachers were transferred to a new school at the end of the training. In the new school, the administration was not informed about the training the teachers had received and was therefore neither prepared nor cooperative in implementing the learning. As a result, the training was not applied in the new schools.

## 5. Discussion

The present study aimed to evaluate the effectiveness of an antibullying intervention specifically designed for Pakistani schools, and implemented in public schools in the rural region of Gilgit-Baltistan. This is the first evidence-based study conducted in the region to assess the outcomes of the intervention. However, it is important to acknowledge that the intervention was not implemented in its entirety as originally planned; therefore, the observed outcomes reflect partial rather than full implementation of the Sohanjana protocol. The intervention involved training teachers and peer mentors, with pre- and post-assessments measuring improvements in knowledge, reductions in bullying incidents and improvements in creating bullying free zones. A notable strength of this study is that the findings are not based solely on the perspective of a single group; rather, conclusions were drawn from reports provided by both staff and students. In addition, the study highlights the challenges faced by researchers in delivering online training and offers recommendations for improving future online training programs. The summary of the overall findings can be found in Table 6.

The intervention outcomes showed moderate post-training improvements, indicating potential gains in participants’ understanding of bullying concepts, consequences, root causes, and effective strategies. These findings are consistent with previous implementations, where both the pilot phase and the first implementation demonstrated the success of the intervention in improving participants’ knowledge [47,48,66]. The intervention consisted of eight teacher trainings and one peer training. In previous implementations, some sessions (one during the piloting phase and three during the first implementation) did not result in significant improvement [48,66]. In contrast, in the current study, statistically significant pre–post improvements were observed across all training modules, including peer training. One possible explanation for this is the limited availability of antibullying programs in the rural area, resulting in lower initial knowledge among teachers, as reported by Siddiqui et al. (2023) [46]. This was not the case in the first implementation, where teachers from elite urban schools already had some basic understanding of bullying, leading to less measurable improvement [48].

Previous research has consistently shown that most interventions are successful in increasing participants’ knowledge about bullying [61]. However, the success rate in reducing actual bullying and victimization varies [48,73,74,75]. In the current study, participants showed improved knowledge of bullying prevention and control strategies; however, the extent to which these strategies were implemented in practice appeared to be more limited. Siddiqui and Schultze-Krumbholz (2026) have reported that even after training is completed, a lack of implementation often persists due to various factors such as unsupportive administration, time constraints, and lack of commitment [48]. A similar pattern was observed in this study, where the final presentation revealed a noticeable weakness in the intensity and consistency of the implementation. These observations suggest that improvements in knowledge may not necessarily translate directly into consistent implementation of learned strategies and highlight the importance of considering contextual and organizational factors when delivering anti-bullying interventions [76].

When teachers shared their experiences during the final presentation, along with photographs and written documentation, many reported that they had organized awareness seminars, held discussions with pupils or installed bullying reporting boxes in their institutions. However, several additional strategies that were addressed in the individual modules could have been implemented to further strengthen antibullying efforts. These include using PE lessons to engage pupils in cooperative games, organizing role-plays or performances during morning assemblies, organizing poster competitions and incorporating stories about bullying into language lessons for reading and reflection, introducing problem solving techniques, emotional management techniques, promoting assertive and communication. For further details on activities that could have been implemented, please refer to previous studies [22,47]. There were challenges in achieving consistent teacher participation in this area [77,78]. Nawab (2020) clearly stated that teachers in rural Pakistan often exhibit reduced interest in professional development, or if they participate in limited opportunities, they rarely make significant changes to their practices [77]. One potential explanation, although not empirically proven, is that the system in which these teachers work lacks effective monitoring and accountability mechanisms. This is supported by studies in developing countries, which suggest that, unlike public school teachers, private school teachers are more accountable to school owners and the community and are more likely to properly implement learned practices [78]. The lack of interest among public sector teachers is also attributed to the absence of recognition or rewards for professional development, which has little impact on their careers, leading them to show minimal concern for these activities [77]. Furthermore, the limited monitoring of how training-related learning is applied in public schools may present a challenge to the sustained implementation of the knowledge and strategies gained during professional development as indicated by studies in Nepal and Bangladesh [79,80]. For future interventions, a stronger encouragement system should be put in place to ensure treatment fidelity as suggested by Siddiqui & Schultze-Krumbholz (2026) [48].

Although the intervention was not implemented as rigorously as planned, post-test results reported by the students showed a significant pre–post reductions in both victimization and perpetration. A statistically significant reduction was also observed in students’ fear of being bullied at school. These results are in line with the first implementation of the intervention, where a decrease-although not always statistically significant-was observed across different grade levels, with a significant decrease in victimization grade 5 students in particular [29]. The previous implementation also reported a significant reduction in physical bullying in rural areas [48]. It was observed that antibullying training programs are rare in Pakistani educational settings including rural contexts [9]. As a result, the new information shared with participants may have raised their awareness of the topic. Exposure to new information and strategies may have contributed to increased awareness among participants [81]; however, the present study cannot determine whether this increased awareness directly contributed to the observed changes in student-reported outcomes. In particular, the involvement of peer mentors may have supported opportunities to discuss and apply antibullying strategies, although this interpretation requires further investigation. The intervention may have reduced bullying by raising empathy, increasing awareness of harm, and making anti-bullying norms more salient, which in turn could lower reported bullying involvement or make bullying seem less prevalent [82,83,84,85]. As highlighted by Siddiqui and Schultze-Krumbholz (2026), the intervention has significant potential to reduce bullying incidents in rural areas [48]. The current findings provide additional preliminary evidence of observed pre–post changes in this context, but controlled studies with larger samples and longer-term follow-up are needed to determine whether these changes can be attributed to the intervention and whether they are maintained over time.

Although student reports indicated a significant pre–post reduction in bullying incidents, staff reports did not support these findings. In fact, in the post-intervention data, there was a slight, although not statistically significant, increase in various forms of bullying. Similar results were seen in the previous implementation, particularly in relation to cyberbullying, where an increase in cases following the intervention were reported [48,86]. This increase was attributed to increased awareness and understanding of different forms of bullying [48]. As this was the first time such a training program had been implemented, it is likely that staff were initially unaware that certain behaviors fell under the category of bullying, resulting in fewer incidents being reported prior to the intervention [48,87]. The increase in reported cases after the intervention suggests that staff became more vigilant and better equipped to recognize bullying behavior. Another possible explanation is shifting baselines, after students learn more about bullying and teachers are trained in prevention, they may evaluate behaviors differently and expect stronger responses, which can change how incidents are perceived or reported [88,89]. Another possible explanation can be that students and teachers often observe bullying in different contexts: students are more likely to witness covert, relational, and out-of-class incidents, whereas teachers mainly observe classroom behavior or incidents brought to their attention; these differences can possibly explain divergent reports [90,91]. Given that not all staff received training or relevant information prior to the pre-intervention data collection, it is recommended that future interventions ensure that staff are introduced to anti-social and bullying behaviors in advance to improve the accuracy and consistency of reported data.

Differences between student and staff reports should be interpreted cautiously, as several factors may contribute to variations in perceived bullying. These may include differences in the instruments or measurement approaches used to assess bullying, sampling variability, differences in reporting thresholds or interpretations of bullying-related behaviors, and genuine differences in how bullying is perceived by students and staff. In addition, differences in the characteristics and contexts of the participating schools may have contributed to variation in reported experiences. Future research should use more directly comparable and, where possible, psychometrically aligned instruments to assess student and staff perceptions of bullying, while also recruiting a larger and more diverse sample of schools to allow these differences to be examined more systematically.

Several studies have already reported that bullying is more prevalent in unstructured areas or places with minimal adult supervision and monitoring [29,92]. In previous implementations of antibullying interventions, the playground was identified as the most common location for bullying, followed by classrooms without teachers present [29]. However, in the current study, the classroom without teacher or staff supervision emerged as the most commonly reported location for bullying. Interestingly, although less but two students (6%) also reported witnessing bullying in the presence of teachers. This may indicate a lack of ability on the part of teachers to intervene [25], difficulty in recognizing or identifying the behavior as unacceptable [46], or even an assumption that the behavior is harmless or playful [93], which has been noted in previous research in Pakistan [23].

It is important to emphasize that while some positive pre–post changes have been reported in the present article regarding the evaluation of the antibullying intervention, the researchers contend that it is also crucial to address the key challenges encountered during the online training programs. The foremost challenge was internet connectivity in rural areas. Although the Education Department of Gilgit Baltistan had made provisions for what appeared to be a high-bandwidth internet connection at the training center, where teachers were invited to participate in online sessions, poor bandwidth and frequent disconnections from the participants’ sites posed significant issues. These technical difficulties not only resulted in time loss but also may have contributed to a decline in trainee motivation, ultimately leading to a failure to complete the training, as reported in previous studies as well [94,95,96]. While internet issues are not uncommon in Pakistan, the recent introduction of a government-imposed firewall has exacerbated the situation, disrupting services even in areas where internet connections had previously been stable. These issues further impeded the effectiveness of online training and overall productivity [97]. Although online instruction has increased after COVID-19, online training in Pakistan continues to face numerous challenges in Pakistan due to technical issues such as poor internet connectivity, outdated software and limited computer literacy, all of which disrupt the teaching and learning process [98]. Key challenges related to online trainings include managing interaction, time management, keeping learners motivated, adapting to new formats, conducting assessments and ensuring equity [99,100,101,102]. In addition, the online delivery of practical skills-traditionally based on interactive face-to-face encounters-can be intimidating for learners used to traditional classroom settings [103]. It should be noted that these internet issues also might have influenced outcomes of the study. Therefore, feasibility of implementing the intervention should be considered separately from the observed pre–post changes. Technical difficulties occasionally interrupted online sessions and may have limited participants’ ability to attend or engage with intervention activities as intended. In combination with variability in attendance and incomplete intervention exposure, these implementation challenges may have influenced the observed outcomes. Therefore, the pre–post findings should be interpreted within the context of the implementation conditions rather than as a direct evaluation of intervention effectiveness independent of implementation feasibility. The experiences from the present study also highlight the importance of considering technological infrastructure and delivery conditions when implementing similar interventions in settings with limited or inconsistent internet connectivity.

One of the key challenges was the reluctance of participants, especially female teachers, to attend afternoon sessions due to family responsibilities or restrictions. Khalid (2024) had previously highlighted that gender norms and regional disparities often limit access to online professional development, particularly for women and individuals in rural areas [104]. Throughout these sessions, the researchers recognized these challenges and made efforts to accommodate the participants as much as possible.

Another issue faced by the researchers was the challenge of data collection through online platforms. Although the trainings were held at a government education building where internet access appeared to be available, trained teachers were required to gather data from students and other staff members using Google Forms at their respective institution. However, neither the staff members nor the students and their parents were cooperative, and many experienced internet connectivity issues, which made data collection a significant challenge. Similar issues are also reported by other national and international studies as well [105,106,107]. As a result, despite expectations of collecting data from hundreds of students, only a few dozen responses were obtained. Khalid (2024) highlighted that the high cost of internet access and digital devices remains a major barrier, particularly for individuals in rural areas who cannot afford these resources [104]. Likewise, many professionals are unable to access the necessary technology, which limits their capacity to fully engage in online professional development opportunities and to facilitate the data collection process for others.

A lack of accountability and interest in implementation was another issue faced by the researchers. Despite attending the trainings, many teachers failed to implement their learnings as expected. Several teachers did not even complete half of the training sessions, and many were absent during the final presentation, where they were supposed to share their experiences of implementing the learned concepts. Nawab (2020) and Mbiti (2016) highlighted that rural public-school teacher in Pakistan often show low interest and accountability in professional development, with little change in their practices [77,78]. However, offering administrative support through financial incentives, promotions, or recognition can encourage participation and lead to improved performance and practice change.

## 6. Limitations and Recommendations

While the study provides valuable insights into the implementation of an online antibullying intervention in Pakistan, several limitations must be acknowledged. The study included relatively small, paired samples of 35 students and 31 staff. No a priori sample size or power calculation was conducted, as participant recruitment was based on the availability and participation of schools and participants within the intervention setting. The small sample may have limited the statistical power to detect changes, particularly where effects were small. Therefore, the analyses should be considered exploratory, and the findings should be interpreted as preliminary evidence of changes observed between the pre- and post-intervention assessments. Larger studies with adequately powered samples are needed to further examine these findings. The use of convenience/purposive sampling may also have introduced selection bias and limits the generalizability of the findings. Attrition is another potential source of selection bias, particularly given the single-group pre–post design. Participants who completed all the trainings have differed from those who attended partial trainings in ways that could have influenced the observed changes. Consequently, the findings may be subject to attrition-related bias and should be considered preliminary rather than interpreted as evidence of causal intervention effects. Therefore, the findings should be interpreted within the context of the participating schools and should not be generalized to rural Pakistan as a whole.

The intervention was not implemented as vigorously as originally planned, mainly due to logistical challenges and participant constraints. Consequently, the observed outcomes reflect partial implementation of the intervention rather than the full Sohanjana protocol, which may have influenced the extent to which the intended outcomes were achieved. Ensuring consistent and thorough implementation, together with closer monitoring and support, would help maximize the impact of the program.

In addition, the short-term nature of the study made it difficult to assess the long-term sustainability and lasting effects of the intervention. Future research should include follow-up assessments several months after the intervention to better understand its long-term impact on behavior and attitudes towards bullying.

Another limitation was the lack of initial information provided to staff prior to the pre-intervention data collection. It is likely that some staff were unfamiliar with the behaviors categorized as bullying, which may have affected the accuracy of the pre-test data. In future implementations, comprehensive training of staff prior to data collection would improve data quality and staff engagement.

Another limitation of the study is the use of a single-group pre–post design without a control group. Although this design allowed us to examine changes in the measured outcomes between the pre-intervention and post-intervention assessments, it does not allow the observed changes to be attributed solely to the intervention. Future studies using controlled or randomized designs would provide stronger evidence regarding the causal effects of the intervention.

One of the main methodological limitations of this study is the use of different scales to measure the variable across respondents, which affects consistency and comparability. Differences in scale sensitivity can lead to discrepancies in how responses are captured, making it difficult to integrate and analyze the data effectively. Different types of measurement can lead to response bias, as participants may interpret the variable differently depending on the scale used. This inconsistency limits the statistical methods available and may affect the reliability of the conclusions drawn. While triangulation increases the validity of the data, the variation in measurement scales poses a challenge in ensuring consistent interpretation. To mitigate this limitation, future research should prioritize standardized measurement approaches or use appropriate data transformation techniques to ensure comparability.

The intervention was implemented at the school level, with participants nested within schools. Although 16 schools initially agreed to participate, complete data were available from only 4 schools for the student sample and 7 schools for the peer-mentor and staff samples. The analyses treated individual participants as the unit of analysis and did not explicitly account for potential clustering by school. Consequently, participants from the same school may have shared contextual characteristics that could have influenced their responses. The small number of schools contributing complete data also limited the feasibility of reliably estimating school-level effects. This issue should therefore be considered when interpreting the exploratory findings, and future studies involving a larger number of schools should incorporate school-level clustering into both the study design and statistical analysis.

Another limitation concerns potential reporting and observer bias. Because some teachers who participated in the intervention were also involved in data collection, their awareness of the intervention and its objectives may have influenced staff-reported responses or the reporting of student outcomes. Although standardized questionnaires and data-collection procedures were used and participants were informed that their responses were collected for research purposes and treated confidentially, social-desirability and observer bias cannot be completely excluded. Therefore, the observed pre–post changes should be interpreted cautiously, and future studies should consider using independent data collectors and, where feasible, multiple sources of outcome assessment.

The study did not include a standardized prospective fidelity checklist or systematic measure of adherence, frequency, duration, and reach for each intervention component. Therefore, a quantitative intervention-fidelity score could not be reliably calculated retrospectively. Future studies should incorporate systematic fidelity monitoring to document which intervention components are implemented, their frequency and duration, and the proportion of the intended participants reached. This would allow the relationship between implementation fidelity and intervention outcomes to be examined more rigorously.

Finally, the reliance on online training presented unique challenges, including limited participation and difficulties in maintaining engagement. Future interventions could benefit from face-to-face training, complemented by ongoing monitoring and support to ensure effective implementation. On-site, paper-based data collection could also help to improve response rates and data accuracy.

By addressing these limitations, future research can build on the current findings and further improve the effectiveness of the program.

## 7. Implications

This study represents the first effort to implement and evaluate an online, contextualized anti-bullying intervention in public rural schools of Pakistan, assessing its impact on reducing bullying and victimization based on feedback from students and staff at participating institutions. Despite challenges related to online training and data collection, the results showed positive outcomes, including a reduction in bullying incidents, victimization, and students’ fear of being bullied at school. While the findings would have been stronger with a higher response rate, they still demonstrate the intervention’s potential. Future improvements could be made by conducting face-to-face sessions, ensuring better monitoring of implementation, and collecting data on-site through paper-based methods. Replicating the study in the future, with the identified challenges addressed and suggested solutions implemented, could yield even more robust results.

## 8. Conclusions

This study represents an initial attempt to implement and examine an online, contextualized anti-bullying intervention in rural Pakistan, addressing an important gap in bullying prevention research in the region. Preliminary pre–post changes were observed in bullying and victimization outcomes from students reports, as well as in students’ perceptions of safety at school, following implementation of the intervention, in addition to the knowledge change among trained teachers and peer mentors; however, no pre–post changes were observed based on teachers reports. The findings are consistent with previous implementations, where improvements in knowledge were observed in addition to reduction in victimization among 5th grade students and reduction in physical bullying in rural schools.

Future implementation should begin with an assessment of infrastructure and technology readiness to identify potential barriers related to internet connectivity, electricity, and device access, with face-to-face or hybrid training considered where fully online delivery is not feasible. Schools should designate an implementation leader, provide protected and context-sensitive training time, and establish formal fidelity-monitoring procedures to document intervention implementation and reach. Offline and paper-based alternatives should be available for data collection where digital access is limited, and staff should receive baseline orientation to ensure a shared understanding of bullying-related behaviors. To support sustainability, continuity procedures should be established when trained teachers transfer schools, alongside ongoing implementation coaching and follow-up support. Finally, larger-scale evaluations involving more schools and stronger comparison conditions are recommended before drawing conclusions about the intervention’s broader effectiveness and scalability.

These findings provide initial information regarding the feasibility of implementing a contextualized anti-bullying intervention in a rural and underserved setting. However, given the single-group pre–post design, variability in intervention exposure and attendance, implementation challenges, and the absence of longer-term follow-up, the findings should be interpreted cautiously and should not be considered evidence of definitive intervention effectiveness or causal impact. Future research using controlled designs, larger samples, independent outcome assessment, and longer-term follow-up is needed to determine whether the observed changes can be attributed to the intervention and whether they are maintained over time. Such research can further inform the development and potential adaptation of scalable anti-bullying interventions for rural and underserved educational settings.

## Figures and Tables

**Figure 1:**
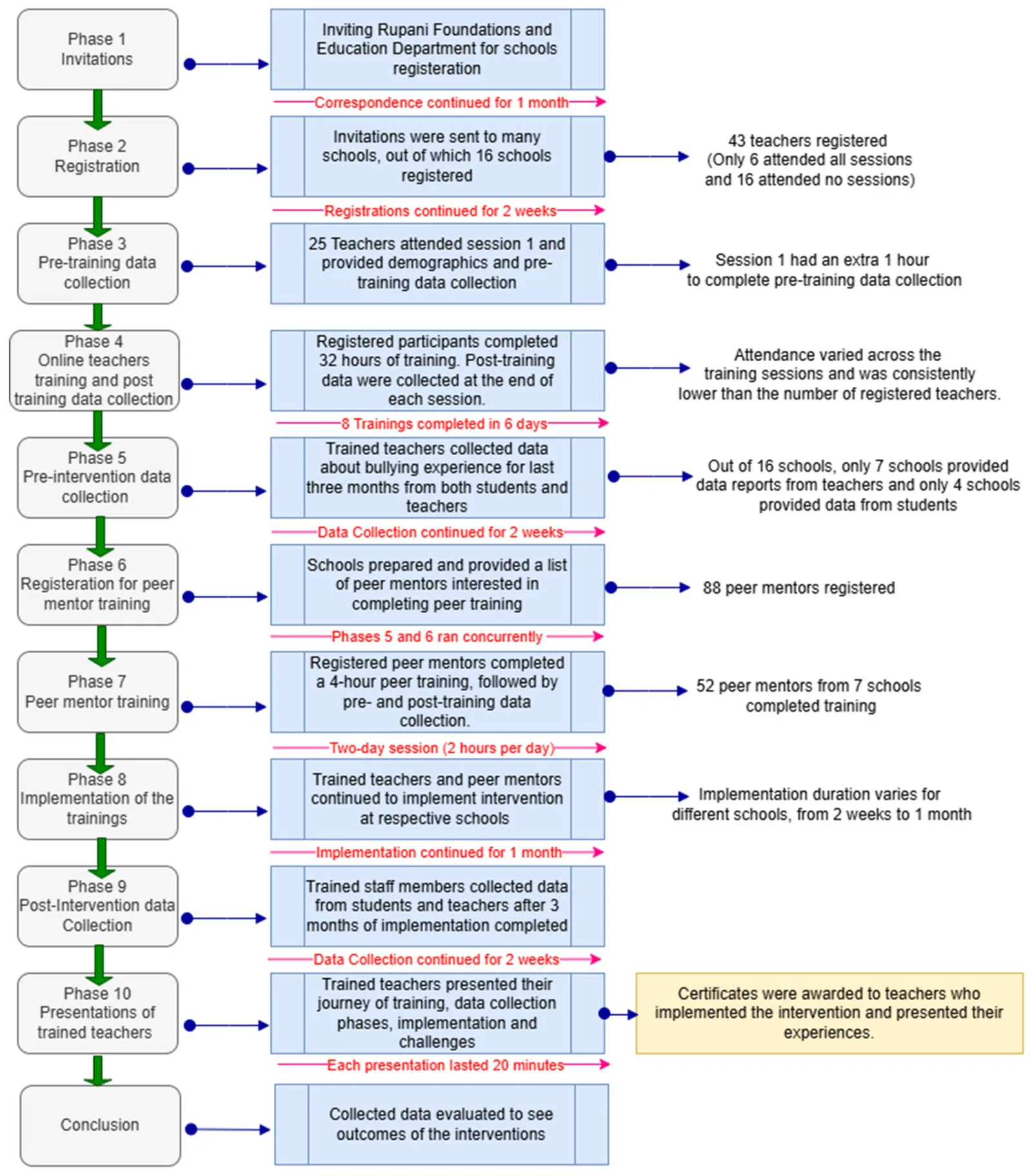
Timeline for the study.

**Figure 2:**
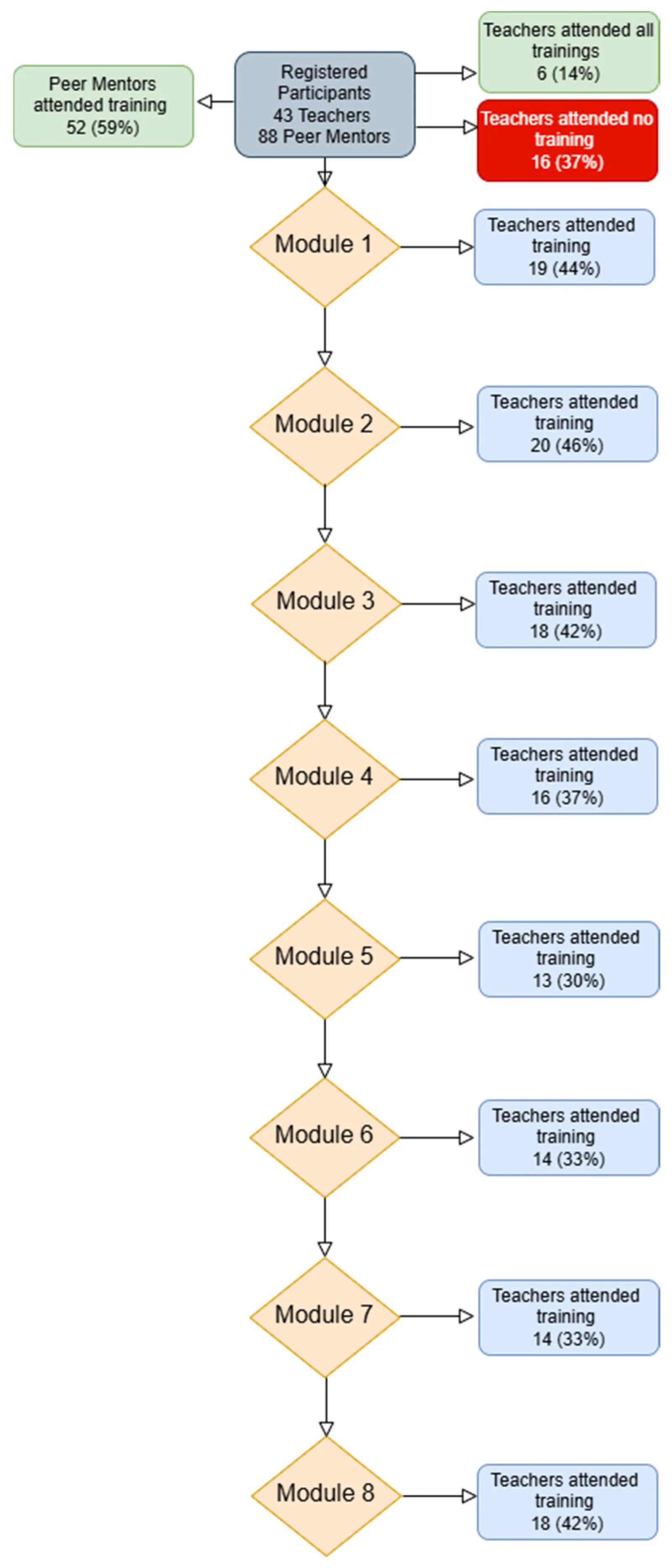
Individual attendance in different training modules.

**Table 1:** Demographics of the Trained Teachers (*n* = 25), Trained Peer Mentors (*n* = 52), Students participating in pre–post survey (*n* = 35) and Staff Members participating in pre–post survey (*n* = 31).

Participants	Gender	Age	Participants and Institutions	Teaching Level	Qualification
Trained Teachers (*n* = 25)	Male = 13 (52%)	30–35 years = 10 (40%)	Institution 1 = 2 (8%)	Primary = 5 (20%)	Graduate = 5 (20%)
			Institution 2 = 3 (12%)		
			Institution 3 = 1 (4%)		
		36–41 years = 5 (20%)	Institution 4 = 3 (12%)		
			Institution 5 = 1 (4%)		
			Institution 6 = 2 (8%)	Secondary = 16 (64%)	Masters = 17 (68%)
			Institution 7 = 1 (4%)		
		42–47 years = 5 (20%)	Institution 8 = 4 (16%)		
	Female = 12 (48%)		Institution 9 = 1 (4%)		
			Institution 10 = 1 (4%)		
		48–53 years = 3 (12%)	Institution 11 = 1 (4%)	Higher Secondary = 4 (16%)	M.Phil./Ph.D. = 3 (12%)
			Institution 12 = 1 (4%)		
			Institution 13 = 1 (4%)		
		54–59 years = 2 (8%)	Institution 14 = 1 (4%)		
			Institution 15 = 1 (4%)		
			Institution 16 = 1 (4%)		
Peer Mentors (*n* = 52)	**Gender**	**Grades**	**Participants and Institutions**	**Age (years)**	
	Boy = 19 (36.5%)	6 = 1 (1.9%)	Institution 3 = 6 (11.5%)	12 = 1 (1.9%)	
		7 = 6 (11.5%)	Institution 10 = 7 (13.5%)	13 = 3 (5.8%)	
		8 = 20 (38.5%)	Institution 13 = 6 (11.5%)	14 = 18 (34.6%)	
	Girl = 33 (63.5%)	9 = 12 (23.1%)	Institution 11 = 12 (23.1%)	15 = 17 (32.7%)	
		10 = 13 (25%)	Institution 2 = 9 (17.3%)	16 = 10 (19.2%)	
			Institution 7 = 7 (13.5%)	17 = 2 (3.8%)	
			Institution 9 = 5 (9.6%)	18 = 1 (1.9%)	
Pre–Post Students (Reported Bullying) (*n* = 35)	**Gender**	**Grades**		**Participants from each Institution**	
	Male = 16 (45.7%)	6 = 3 (8.6%)		Institution 9 = 9 (25.7%)	
		7 = 1 (2.9%)		Institution 7 = 7 (20%)	
		8 = 17 (48.6%)		Institution 10 = 7 (20%)	
	Female = 19 (54.3%)	9 = 9 (25.7%)		Institution 2 = 12 (34.3%)	
		10 = 5 (14.3%)			
Pre–Post Staff Members Reported Bullying (*n* = 31)	**Gender**	**Age**	**Participants and Institutions**	**Designation**	**Experience in Education Field**
	Male = 10 (32.3%)	21–25 years = 3 (9.7%)	Institution 9 = 3 (9.7%)	Teacher = 28 (90.3%)	Less than 5 years = 1 (3.2%)
		26–30 years = 10 (32.3%)	Institution 2 = 11 (35.5%)		5–10 years = 13 (41.9%)
		31–35 years = 9 (29%)	Institution 7 = 3 (9.7%)	Administrator = 1 (3.22%)	11–15 years = 8 (25.8%)
	Female = 21 (67.7%)	36–40 years = 5 (16.1%)	Institution 3 = 5 (16.1%)		16–20 years = 4 (12.9%)
		41–45 years = 1 (3.2%)	Institution 10 = 7 (22.6%)	Sports Staff = 2 (6.45%)	More than 20 years = 5 (16.1%)
		46–50 years = 2 (6.5%)			
		More than 50 years = 1 (3.2%)			

**Table 2:** Pre–Post Test (Wilcoxon Signed Rank Test).

Module	No. of Participants	Pre–Post Test Sum	Mean Value	Standard Deviation	Wilcoxon (Z-Value)	*p*-Value	Holm-Adjusted *p*	Significant After Adjustment	Frequencies (Post Test Sum-Pre Test Sum)	N	Effect Size (r)
Module I	19	Pre-test Sum	4.5263	1.1723	−2.634 ^x^	0.008	0.018	Yes	Negative Differences ^a^	3 ^a^	0.604
		Post-test Sum	5.5789	0.96124					Positive Differences ^b^	14 ^b^	
									Ties ^c^	2 ^c^	
Module II	20	Pre-test Sum	4.1500	1.3088	−2.741 ^x^	0.006	0.018	Yes	Negative Differences ^a^	3 ^a^	0.613
		Post-test Sum	5.8000	1.54238					Positive Differences ^b^	15 ^b^	
									Ties ^c^	2 ^c^	
Module III	18	Pre-test Sum	4.111	1.323	−3.001 ^x^	0.003	0.007	Yes	Negative Differences ^a^	1 ^a^	0.707
		Post-test Sum	5.5556	1.04162					Positive Differences ^b^	12 ^b^	
									Ties ^c^	5 ^c^	
Module IV	16	Pre-test Sum	2.3125	1.537	−3.090 ^x^	0.002	0.007	Yes	Negative Differences ^a^	0 ^a^	0.773
		Post-test Sum	4.5000	1.67332					Positive Differences ^b^	12 ^b^	
									Ties ^c^	4 ^c^	
Module V	13	Pre-test Sum	2.4615	1.9414	−3.188 ^x^	0.001	0.010	Yes	Negative Differences ^a^	0 ^a^	0.884
		Post-test Sum	6.4615	1.05003					Positive Differences ^b^	13 ^b^	
									Ties ^c^	0 ^c^	
Module VI	14	Pre-test Sum	2.7143	1.898	−2.412 ^x^	0.016	0.018	Yes	Negative Differences ^a^	2 ^a^	0.645
		Post-test Sum	4.0000	1.51911					Positive Differences ^b^	11 ^b^	
									Ties ^c^	1 ^c^	
Module VII	14	Pre-test Sum	2.8571	1.61041	−3.198 ^x^	0.001	0.007	Yes	Negative Differences ^a^	0 ^a^	0.855
		Post-test Sum	5.8571	1.74784					Positive Differences ^b^	13 ^b^	
									Ties ^c^	1 ^c^	
Module VIII	18	Pre-test Sum	2.444	0.983	−3.533 ^x^	<0.001	<0.001	Yes	Negative Differences ^a^	0 ^a^	0.833
		Post-test Sum	5.1111	1.45072					Positive Differences ^b^	16 ^b^	
									Ties ^c^	2 ^c^	
Peer Training Module	52	Pre-test Sum	3.5192	1.59029	−3.587 ^x^	<0.001	<0.001	Yes	Negative Differences ^a^	9 ^a^	0.498
		Post-test Sum	4.4231	1.80789					Positive Differences ^b^	28 ^b^	
									Ties ^c^	15 ^c^	

a. PostTestSum < PreTestSum. b. PostTestSum > PreTestSum. c. PostTestSum = PreTestSum. x. Based on negative ranks.

**Table 3:** Pre–Post Victimization and Perpetration Comparison (Wilcoxon Signed Rank Test) (Students Report, *n* = 35).

Variable	No. of Participants	Pre–Post Test	Cronbach Alpha	Mean Value	Standard Deviation	Wilcoxon (Z-Value)	*p*-Value	Holm-Adjusted *p*	Significant After Adjustment	Frequencies (Post Score Sum-Pre Score Sum)	N	Effect Size (r)
Victimization No. of items = 10	35	Pre-Victimization	0.809	1.6314	0.65474	−3.838 ^x^	<0.001	<0.001	Yes	Negative Differences ^a^	28 ^a^	0.65
		Post- Victimization	0.793	1.1514	0.31843					Positive Differences ^b^	3 ^b^	
										Ties ^c^	4 ^c^	
Perpetration No. of items = 10	35	Pre-Perpetration	0.673	1.2829	0.36420	−3.049 ^x^	0.002	0.002	Yes	Negative Differences ^a^	20 ^a^	0.52
		Post- Perpetration	0.762	1.0743	0.20050					Positive Differences ^b^	4 ^b^	
										Ties ^c^	11 ^c^	

a. PostTestSum < PreTestSum. b. PostTestSum > PreTestSum. c. PostTestSum = PreTestSum. x. Based on negative ranks.

**Table 4:** Pre–post comparison of attitudes of teachers, students, and parents (Wilcoxon Signed Rank Test).

Variable	No. of Participants	Pre–Post Intervention	Mean Value	Standard Deviation	Wilcoxon (Z-Value)	*p*-Value	Holm-Adjusted *p*	Significant After Adjustment	Frequencies (Post Test Sum-Pre Test Sum)	N	Effect Size (r)
Institutional Staff Attitude	35	Pre-intervention	3.8571	1.53667	−1.121 ^x^	0.262	0.786	No	Negative Differences ^a^	15 ^a^	0.189
		Post-intervention	3.4000	1.76901					Positive Differences ^b^	8 ^b^	
									Ties ^c^	12 ^c^	
Students Attitude	35	Pre-intervention	4.0571	1.34914	−1.678 ^x^	0.093	0.744	No	Negative Differences ^a^	17 ^a^	0.28
		Post- intervention	3.5143	1.66930					Positive Differences ^b^	6 ^b^	
									Ties ^c^	12 ^c^	
Parents/ Guardians Attitude	35	Pre-intervention	1.6000	0.91394	−0.846 ^x^	0.398	0.786	No	Negative Differences ^a^	11 ^a^	0.143
		Post- intervention	1.4286	0.91670					Positive Differences ^b^	6 ^b^	
									Ties ^c^	18 ^c^	
Class Teacher Attitude	35	Pre-intervention	3.3714	1.75039	−1.119 ^x^	0.263	0.786	No	Negative Differences ^a^	16 ^a^	0.189
		Post- intervention	2.9429	1.74799					Positive Differences ^b^	9 ^b^	
									Ties ^c^	10 ^c^	
Liking of Institution	35	Pre-intervention	4.6286	1.05957	−1.91 ^x^	0.055	0.440	No	Negative Differences ^a^	13 ^a^	0.323
		Post- intervention	4.2000	1.27879					Positive Differences ^b^	3 ^b^	
									Ties ^c^	19 ^c^	
Tendency towards bullying	35	Pre-intervention	3.7143	1.80801	−1.232 ^x^	0.218	0.786	No	Negative Differences ^a^	10 ^a^	0.208
		Post- intervention	4.2286	1.49678					Positive Differences ^b^	15 ^b^	
									Ties ^c^	10 ^c^	
Reactions towards bullying	35	Pre-intervention	3.3714	2.22401	−0.396 ^x^	0.692	0.786	No	Negative Differences ^a^	14 ^a^	0.067
		Post- intervention	3.0857	2.36856					Positive Differences ^b^	11 ^b^	
									Ties ^c^	10 ^c^	
Fear of getting bullied	35	Pre-intervention	3.0571	1.95453	−2.806 ^x^	0.005	0.045	Yes	Negative Differences ^a^	19 ^a^	0.5
		Post- intervention	1.8000	1.18322					Positive Differences ^b^	9 ^b^	
									Ties ^c^	7 ^c^	
Empathy	35	Pre-intervention	3.2857	1.15227	−1.368 ^x^	0.171	0.786	No	Negative Differences ^a^	10 ^a^	0.231
		Post- intervention	2.9429	1.32716					Positive Differences ^b^	7 ^b^	
									Ties ^c^	18 ^c^	

a. PostTestSum < PreTestSum. b. PostTestSum > PreTestSum. c. PostTestSum = PreTestSum. x. Based on negative ranks.

**Table 5:** Pre–Post Different forms of Bullying Comparison–Staff Reports (Wilcoxon Signed Rank Test).

Variable	No. of Participants	Pre–Post Score	Cronbach Alpha	Mean Value	Standard Deviation	Wilcoxon (Z-Value)	*p*-Value	Frequencies (Post Score Sum-Pre Score Sum)	N	Effect Size (r)
Cyberbullying No. of items = 17	31	Pre-test Sum	0.886	2.6983	0.56457	−1.206 ^x^	0.228	Negative Differences ^a^	11 ^a^	0.217
		Post-test Sum	0.924	2.8501	0.64881			Positive Differences ^b^	20 ^b^	
								Ties ^c^	0 ^c^	
Emotional/Social Bullying No. of items = 9	31	Pre-test Sum	0.857	3.2652	0.64258	−0.942 ^x^	0.346	Negative Differences ^a^	16 ^a^	0.169
		Post-test Sum	0.914	3.1362	0.74132			Positive Differences ^b^	13 ^b^	
								Ties ^c^	2 ^c^	
Verbal Bullying No. of items = 7	31	Pre-test Sum	0.788	3.1982	0.59782	−0.670 ^x^	0.503	Negative Differences ^a^	14 ^a^	0.120
		Post-test Sum	0.908	3.2535	0.78047			Positive Differences ^b^	16 ^b^	
								Ties ^c^	1 ^c^	
Physical Bullying No. of items = 7	31	Pre-test Sum	0.869	2.9539	0.62530	−0.115 ^x^	0.909	Negative Differences ^a^	13 ^a^	0.021
		Post-test Sum	0.922	2.9631	0.72559			Positive Differences ^b^	13 ^b^	
								Ties ^c^	5 ^c^	

a. PostTestSum < PreTestSum. b. PostTestSum > PreTestSum. c. PostTestSum = PreTestSum. x. Based on negative ranks.

**Table 6:** Summary of the findings.

Variable	No. of Items	Participant Group	No. of Participants Responded	Outcomes
Knowledge Change-Module 1	8 closed-ended questions	Staff completed training	19	Significant and positive improvement
Knowledge Change-Module 2	4 closed-ended questions and 2 open-ended questions	Staff completed training	20	Significant and positive improvement
Knowledge Change-Module 3	4 closed-ended questions and 3 open-ended questions	Staff completed training	18	Significant and positive improvement
Knowledge Change-Module 4	3 closed-ended questions and 2 open-ended questions	Staff completed training	16	Significant and positive improvement
Knowledge Change-Module 5	7 closed-ended questions and 1 open-ended question	Staff completed training	13	Significant and positive improvement
Knowledge Change-Module 6	5 closed-ended questions and 3 open-ended questions	Staff completed training	14	Significant and positive improvement
Knowledge Change-Module 7	6 closed-ended questions and 2 open-ended questions	Staff completed training	14	Significant and positive improvement
Knowledge Change-Module 8	6 closed-ended questions and 1 open-ended question	Staff completed training	18	Significant and positive improvement
Knowledge change-Peer Mentor Training	6 closed-ended questions and 1 open-ended question	Trained Peer Mentors	52	Significant and positive improvement
Victimization	10	Students of schools where intervention implemented	35	Significant reduction
Perpetration	10	Students of schools where intervention implemented	35	Significant reduction
Institutional Staff Attitude	1	Students of schools where intervention implemented	35	No significant change
Students Attitude	1	Students of schools where intervention implemented	35	No significant change
Parents/ Guardians Attitude	1	Students of schools where intervention implemented	35	No significant change
Class Teacher Attitude	1	Students of schools where intervention implemented	35	No significant change
Liking of Institution	1	Students of schools where intervention implemented	35	No significant change
Tendency towards bullying	1	Students of schools where intervention implemented	35	No significant change
Reactions towards bullying	1	Students of schools where intervention implemented	35	No significant change
Fear of getting bullied	1	Students of schools where intervention implemented	35	Significantly Reduced
Empathy	1	Students of schools where intervention implemented	35	No significant change
Cyberbullying	17	Staff members of schools where intervention implemented	31	No significant change
Emotional/Social bullying	9	Staff members of schools where intervention implemented	31	No significant change
Verbal bullying	7	Staff members of schools where intervention implemented	31	No significant change
Physical bullying	7	Staff members of schools where intervention implemented	31	No significant change

## Data Availability

The findings derived from the dataset have already been reported in the manuscript. The data supporting the findings of this study are available from the corresponding author upon reasonable request, subject to applicable ethical, confidentiality, and data-protection requirements. The dataset is not publicly available due to these restrictions.
